# OR11-004 - IL-1, IL-18 and cell death in NLRP3 driven disease

**DOI:** 10.1186/1546-0096-11-S1-A193

**Published:** 2013-11-08

**Authors:** L Broderick, SD Brydges, MD McGeough, CA Pena, JL Mueller, HM Hoffman

**Affiliations:** 1 University of California San Diego, La Jolla, USA; 2 Rady Children's Hospital, San Diego, USA; 3 Ludwig Institute of Cancer Research, San Diego, USA

## Introduction

Missense mutations in cryopyrin (NLRP3) result in a hyperactive inflammasome that drives overproduction of the pro-inflammatory cytokines interleukin-1b (IL-1b) and IL-18. Mice expressing mutations associated with familial cold autoinflammatory syndrome (FCAS) or Muckle-Wells syndrome (MWS) exhibit severe, spontaneous inflammation, early death, and hyperresponsiveness to stimuli *in vitro*. Abrogating IL-1 signaling either genetically or pharmacologically results in modest improvement of life expectancy in murine CAPS, but clearly indicates a role for players in addition to IL-1b.

## Objectives

To examine the role of other caspase-1 dependent mediators, namely IL-18, in the context of inflammasome-mediated disease.

## Methods

Mice heterozygous for the A350V MWS mutation or the L351P NOMID mutation were bred to IL-1R-/- or IL-18R-/- knockout mice, and weighed and assessed daily. Bone marrow macrophages and peritoneal cells were evaluated *in vitro* with inflammatory stimuli. Peripheral blood was drawn for complete blood counts and serum cytokine analyses. Pathology was examined in both young and old mice. Bone marrow transplant experiments were used to elucidate the role of cellular signaling compartments.

## Results

Similar to IL-1b, hematopoietic cells derived from our mutant mice and monocytes from FCAS patients hyper-secrete IL-18, in response to low amounts of inflammatory stimuli or cold temperature. Breeding *Nlrp3* mutations onto an IL-18R null background resulted in partial phenotypic rescue that abolished skin and visceral disease in young mice, and normalized serum cytokines to a greater extent than breeding to IL-1R null mice. However, significant systemic inflammation developed in aging *Nlrp3* mutant IL-18R null mice, implicating a role for pyroptosis, a caspase-1 mediated form of cell death. Bone marrow transplant studies demonstrate that hematopoietic cells are driving disease in murine CAPS but signaling requirements differ between IL-1 and IL-18.

## Conclusion

These studies demonstrate a previously underappreciated role for IL-18 signaling in murine CAPS pathogenesis. We also confirm our previous findings that CAPS is inflammasome-dependent by demonstrating that intact caspase-1 is required for disease, yet other downstream mechanisms besides IL-18 and IL-1b mediated inflammation are involved in this autoinflammatory syndrome constellation. Our results may have important implications for patients with CAPS and residual disease and emphasize the need to explore other NLRP3 mediated pathways and the potential for inflammasome targeted therapy.

## Competing interests

L. Broderick: None declared, S. Brydges: None declared, M. McGeough: None declared, C. Pena: None declared, J. Mueller: None declared, H. Hoffman Consultant for: Regeneron, Novartis, Sobi Pharmaceuticals.

